# Blood Cells Far from Equilibrium: Redox Adaptation in Health and Disease

**DOI:** 10.3390/antiox15060723

**Published:** 2026-06-05

**Authors:** Alkmini T. Anastasiadi, Angelo D’Alessandro

**Affiliations:** 1 Department of Biochemistry, School of Medicine, University of Patras, 26504 Patras, Greece; 2 Department of Biochemistry and Molecular Genetics, Anschutz Medical Campus, University of Colorado Denver, Aurora, CO 80045, USA

What does it actually mean for a blood cell to be ‘in balance’? In biology, balance is often mistaken for stability. However, living systems exist far from thermodynamic equilibrium, continuously consuming energy to maintain organization while adapting to ever-changing internal and external conditions. In this sense, homeostasis is not a fixed destination but a dynamic process—a moving target shaped by genetics, metabolism, disease, and environment. Equally important, optimal function rarely lies at either extreme. Too little stress can be as harmful as too much, and resilience often emerges within a narrow adaptive window between deficiency and excess. The studies collected in this Special Issue build on the foundations of the first edition [1] and suggest that blood cell homeostasis is best understood not as a static state but as the capacity to continuously adapt to changing conditions.

Although the antioxidant systems of RBCs have been extensively characterized [2,3], an important question remains: does appearing “adequate” at baseline actually mean cells are resilient under stress? Oliveira and colleagues [Contribution 1] address this in the context of Fanconi anemia (FA), a rare bone marrow failure disorder marked by hypersensitivity to alkylating agents and chronic redox imbalance. At baseline, FA RBCs showed paradoxically elevated antioxidant enzyme activity, suggesting a state of compensatory preconditioning. Yet, under oxidative challenge, these defenses rapidly collapsed, while healthy control cells remained stable. The findings portray a fragile equilibrium: one that functions well under ordinary conditions but fails when additional stress is introduced. Approaching the problem from a bioengineering perspective, Langlands, Shoemark and Toye [Contribution 2] demonstrated that culture-derived reticulocytes can be produced with enhanced antioxidant capacity through lentiviral overexpression of peroxiredoxin and glutathione peroxidase isoforms. These engineered reticulocytes displayed greater antioxidant protein levels, providing proof of concept for manufacturing RBCs with improved oxidative resilience, an advance with potential implications for transfusion medicine and diseases with chronic oxidative burden.

Glucose-6-phosphate dehydrogenase (G6PD) deficiency is the most prevalent human enzymopathy, affecting over 500 million individuals worldwide [4]. Traditionally, reduced pentose phosphate pathway activity has been associated with oxidative fragility and impaired physical performance. However, Cendali et al. [Contribution 3] challenged this assumption. In humanized mice carrying the African G6PD variant, treadmill performance unexpectedly improved. Multi-omics profiling across the blood, muscle, spleen, kidney, and liver revealed widespread metabolic adaptation, including elevated glycolysis, enhanced fatty acid oxidation, increased mitochondrial activity in muscle, and accelerated RBC turnover. Rather than simply compensating for enzyme deficiency, the organism underwent a complete systemic remodeling that ultimately resulted in a net gain in performance. This finding reframes G6PD deficiency as a driver of adaptive plasticity, not merely a metabolic liability to be overcome. This study highlights how single-gene defects can trigger systemic remodeling across tissues and pathways, emphasizing the importance of integrated multi-omics approaches in understanding complex biological phenotypes [5].

Platelet biology also emerged as a major focus. Han, Utsumi and Chung [Contribution 4] examined how reactive oxygen species (ROS) contribute to collagen-induced platelet activation. Using electron spin resonance spectroscopy, they monitored ROS generation in real time both in vitro and in vivo. Collagen stimulation led to rapid, dose-dependent increases in ROS production that closely paralleled platelet aggregation and reduced survival in murine models. Importantly, both enzymatic and non-enzymatic antioxidants significantly attenuated these effects, establishing a rational basis for antioxidant intervention in thrombotic cardiovascular disease [6]. The broader landscape of platelet redox regulation, including the enzymatic sources of ROS, the antioxidant systems that counterbalance them, and pharmacological strategies for modulating this balance, is comprehensively reviewed by Dionisio, Zheng, and Cancelas [Contribution 5], providing a systematic framework inspired by mechanistic findings.

The longstanding view of circulating blood cells as passive carriers or effectors continues to evolve [7,8]. Two contributions in this Special Issue reinforce the idea that blood cells actively shape disease processes in clinically meaningful ways. Yang and colleagues [Contribution 6] showed that selectively targeting arginase 1, but not arginase 2, protected mice from myocardial ischemia–reperfusion injury, with RBCs acting as the primary mediators of protection. The mechanism depended on nitric oxide signaling and remained effective in both sexes. Remarkably, the protective effect could also be restored in type 2 diabetic animals through ARG1 knockdown, identifying erythrocyte arginase 1 as a promising therapeutic target in cardiovascular disease associated with metabolic dysfunction [9,10]. Chang and colleagues [Contribution 7] addressed the immunological consequences of RBC transfusion. Alloimmunization remains a major challenge in chronically transfused patients, and there are currently no pharmacological preventive therapies available [11]. The authors demonstrated that activating the Nrf2 pathway using CDDO-imidazole suppressed inflammation-driven anti-KEL antibody production in mice through the inhibition of type 1 interferon signaling in macrophages. These findings suggest that Nrf2, traditionally viewed as the master regulator of antioxidant gene expression [12], may also function as an immunological checkpoint at the transfusion interface, opening up new possibilities for preventing transfusion-related immune complications.

The immunological dimension of redox biology extends even into ethnomedicine [13]. Matano and Patra [Contribution 8] reviewed the potential role of *Annona muricata* (soursop) in modulating hematopoiesis. Bioactive compounds within the plant, including flavonoids, polyphenols, and acetogenins, have demonstrated antioxidant, anti-inflammatory, and immunomodulatory effects in preclinical models. The authors propose that these activities may help preserve hematopoietic stem and progenitor cell function under conditions of oxidative or inflammatory stress. Importantly, the review carefully distinguishes between preliminary experimental evidence and true clinical applicability, while outlining the rigorous studies still needed to bridge that gap.

Collectively, the studies in this Special Issue challenge the notion of blood cell homeostasis as a static state. Instead, they portray blood cells as dynamic adaptive systems that continuously navigate competing oxidative, metabolic, immune, and mechanical pressures. From erythrocytes and platelets to hematopoietic progenitors, health emerges not from the absence of stress but from the capacity to respond appropriately to it. In many ways, these studies echo Aristotle’s concept of the Golden Mean [14]: biological function is optimized not at the extremes of deficiency or excess but within a dynamic adaptive space between them. Understanding the mechanisms that define these adaptive boundaries may ultimately prove more important than defining homeostasis itself. Crucially, the contributions collected here signal a broader transition in the field: from describing how blood cells adapt to redox and metabolic stress toward deliberately harnessing those mechanisms for therapeutic gain, whether by engineering cells with enhanced resilience, targeting specific enzymatic nodes to restore redox balance, or repurposing transcriptional regulators as immunological checkpoints. This shift from characterization to intervention may define the next chapter of blood cell redox biology.
